# Radical Scavenging Activities of Novel Cationic Inulin Derivatives

**DOI:** 10.3390/polym10121295

**Published:** 2018-11-22

**Authors:** Yuan Chen, Yingqi Mi, Jingjing Zhang, Fang Dong, Qing Li, Naiyun Ji, Zhanyong Guo

**Affiliations:** 1 Key Laboratory of Coastal Biology and Bioresource Utilization, Yantai Institute of Coastal Zone Research, Chinese Academy of Sciences, Yantai 264003, China; yuanchen@yic.ac.cn (Y.C.); yqmi@yic.ac.cn (Y.M.); jingjingzhang@yic.ac.cn (J.Z.); fdong@yic.ac.cn (F.D.); qli@yic.ac.cn (Q.L.); 2 University of Chinese Academy of Sciences, Beijing 100049, China

**Keywords:** cationic inulin derivatives, free radicals, radical scavenging activity, amino-pyridine group

## Abstract

Many saccharides are attractive targets for biomaterial applications, due to their abundance, biocompatibility, and biodegradability. In this article, a synthesis process of 6-*N*-substituted cationic inulin derivatives, including 6-pyridyl-6-deoxyinulin bromide (PIL), 6-(2-amino-pyridyl)-6-deoxyinulin bromide (2APIL), 6-(3-amino-pyridyl)-6-deoxyinulin bromide (3APIL), 6-(4-amino-pyridyl)-6-deoxyinulin bromide (4APIL), 6-(2,3-diamino-pyridyl)-6-deoxyinulin bromide (2,3DAPIL), 6-(3,4-diamino-pyridyl)-6-deoxyinulin bromide (3,4DAPIL), and 6-(2,6-diamino-pyridyl)-6-deoxyinulin bromide (2,6DAPIL) was described. The C_6_-OH of inulin was first activated by PPh_3_/N-bromosuccinimide (NBS) bromination. Then, pyridine and different kinds of amino-pyridine groups (different position and different numbers of amino) were grafted onto inulin, respectively, via nucleophilic substitution. Then, we confirmed their structure by Fourier transform infrared spectroscopy (FT-IR) and nuclear magnetic resonance (NMR) spectroscopy. After this, their radical scavenging activities against hydroxyl radical and diphenylpicryl phenylhydrazine (DPPH) radical were tested in vitro. Each derivative showed a distinct improvement in radical scavenging activity when compared to inulin. The hydroxyl-radical scavenging effect decreased in the following order: 3APIL > PIL > 3,4DAPIL > 4APIL > 2,3DAPIL > 2,6DAPIL > 2APIL. Amongst them, 3APIL revealed the most powerful scavenging effect on hydroxyl radicals, as well as DPPH radicals. At 1.6 mg/mL, it could completely eliminate hydroxyl radicals and could clear 65% of DPPH radicals. The results also showed that the steric hindrance effect and the substitute position of the amino group had an effect on the radical scavenging activity. Moreover, the application prospects of inulin derivatives as natural antioxidant biomaterials are scientifically proven in this paper.

## 1. Introduction

Free radicals and antioxidants have been a hot topic for both scientists and the public. For aerobic organisms, oxygen is an essential element that is mainly consumed for mitochondrial respiration to generate energy, but under certain circumstances (oxidative stress), it can cause oxidative damage in cells [1,2,3]. Oxidative damage has a negative influence on cells and tissues, including the molecules that are essential to life, such as DNA, RNA, enzymes, and other biomolecules, leading to numerous disorders, such as cancer, cardiovascular disease, and heart disease, etc., and they are considered to be an important factor for premature aging [4,5]. This damage of oxygen is directly affected by molecules called free radicals, which exist independently with one or more unpaired electrons. They are so reactive that they can cause chain reactions. Free radicals take electrons from other elements or molecules to maintain their own stabilization, leading to some acute and chronic disorders [6,7]. The role of free radicals in many diseases, for instance, Alzheimer’s disease and acute and chronic liver diseases, has been well demonstrated [8,9,10,11]. Radical scavenging activity is considered as an important index for evaluating the antioxidant activity of medicines. 

In the past few decades, the design of polymers for biomaterial applications has brought about widespread attention [12,13,14]. Polysaccharides, such as chitosan and cellulose, are already widely used because of their biocompatibility, biodegradability, and abundance. As a kind of natural plant fructan, inulin was widely discovered in more than 36,000 plant species, as well as in some microorganisms. Commercially, inulin is mainly extracted from chicory and Jerusalem artichoke. Inulin cannot be digested in the small bowel, as it consists of fructofuranose and it is bonded by *β*- (2-1) glycosidic linkages, but it can be fermented by microflora in the large intestine. As a result, inulin is easily dissolved in water, and it exhibits numerous health benefits, including hypoglycemic action, promoting mineral absorption, and reducing the risk of gastrointestinal diseases, etc. [15]. Inulin itself has been extensively used to replace fat or sugar in food industry, and it has unique processing characteristics that have applications in improving the texture of low-fat food [16]. Moreover, inulin finds some new applications in such fields as drug carriers, effluent treatment, biofuels, and tissue engineering scaffolds, etc. [17,18,19,20]. However, it is comparatively insufficient when compared to the utilization of other polysaccharides, such as chitosan, especially when taking the excellent physicochemical properties and unique physiological activities of inulin, as revealed in numerous investigations, into account [21]. Further developments in the technology of producing inulin and other renewable resources, as well as people’s urgent need for green development, will certainly result in a greater research focus on chemical modifications, and on the use of modified natural products in the industry [22]. However, inulin exhibits some antioxidant activities; for instance, the inhibition percentages of inulin against hydroxyl radicals and H_2_O_2_ are about 50% and 40%, respectively, at 1 mg/mL [23]. However, its application as an antioxidant biomaterial is not strong enough. Thus, performing optimal structural modifications of inulin could help to improve its bioactivity, and accordingly, it makes inulin more feasible as a type of antioxidant bio-material. It is well known that the structures of molecules can affect their functions. In recent years, many research efforts have been carried out for the chemical structural modification of natural polysaccharides, in order to gain different advantages, such as improving their solubility, or enhancing their biological activity. For example, many strategies, such as quaternization, carboxylation, phosphorylation, and sulfation have been applied to enhance the water solubility of chitosan, and the biological activities of these derivatives have been improved accordingly [24,25,26]. Zhang et al. modified inulin with octenyl succinic anhydride, and the derivative was able to completely inactivate *Escherichia coli* and *Staphylococcus aureus* at concentrations of 1% and 0.5%, respectively [27]. Iemma et al. synthesized catechin–inulin conjugates by an eco-friendly grafting procedure, and showed that they conferred 74% inhibition against diphenylpicryl phenylhydrazine (DPPH) radicals [28]. Guo et al. modified the structure of starch by different chain lengths of aliphatic alcohol, and analyzed the relationship between the structure and the antifungal activity of these derivatives. They found that the antifungal activities were obviously affected by the structure of these starch derivatives [29].

Stevens provides a relatively integral overview of the chemical modification of inulin and its probable industrialized applications [22]. These modifications include esterification, acylation, etherification, methylation, carboxymethylation, and cationic modification [22]. Among them, cationic polysaccharide derivatives have been designed for a number of applications, ranging from anti-bacterial agents to gene delivery vectors and for sewage treatment [30,31,32,33]. It was reported that the antioxidant capacity of chitosan is not only influenced by hydroxyl groups, but also by amidogen [34]. Furthermore, the antioxidant activity is affected by the form of amidogen and quaternized chitosan with a high positive charge density might account for the best antioxidant activity against hydroxyl radicals [35]. It is reasonable to assume that the introduction of an amino group could help to enhance its biological activity, as well as increasing the density of the positive charge of inulin and promote its application values. In the field of polysaccharide modification, 6-tosylate or 6-halide variants of polysaccharides are regarded as key intermediates that can be replaced by multiple nucleophiles to obtain deoxy-polysaccharide derivatives, such as amino derivatives and cationic derivatives [36,37]. Heteroaromatic compounds, such as pyridine and imidazole, have been grafted onto each kind of polysaccharides. The synthetic strategy was optimized by researchers to meet efficient, simple, and economic purposes [38,39,40,41]. Previously, our team succeeded in the synthesis of *N*-(aminoethyl) inulin, 6-amino-6-deoxy inulin, and 6-azido-inulin for “click” reactions [42,43,44]. In the following study, we attempt to develop novel cationic inulin derivatives modified with pyridine and amino-pyridine groups, and to test their radical scavenging activity, in order to provide an efficient way to prepare novel natural antioxidant biomaterials.

## 2. Materials and Methods

### 2.1. Materials

Inulin that was extracted from chicory was purchased from Haoyuan Biological Technology Co., Ltd. (Xi’an, China) and its average degree of polymerization (DP) was around 20. Pyridine, 2-aminopyridine, 3-aminopyridine, 4-aminopyridine, 2,3-diaminopyridine, 3,4-diaminopyridine, 2,6-diaminopyridine, phenazine methosulfate (PMS), nicotinamide adenine dinucleotide (NADH), and nitro blue tetrazolium (NBT) were purchased from Sigma-Aldrich Chemical Corp. (Shanghai, China). Triphenylphosphine (Ph_3_P), *N*-bromosuccinimide (NBS), acetic anhydride (Ac_2_O), *N*,*N*-dimethylacetamide (DMF), dimethyl sulfoxide (DMSO), acetone, diethyl ether, and ethanol were all purchased from Sinopharm Chemical Reagent Co., Ltd (Shanghai, China). All of the reagents were used without other modifications.

### 2.2. Synthesis of Inulin Derivatives 

#### 2.2.1. Synthesis of 6-Bromo-6-deoxyinulin

**Scheme 1:**
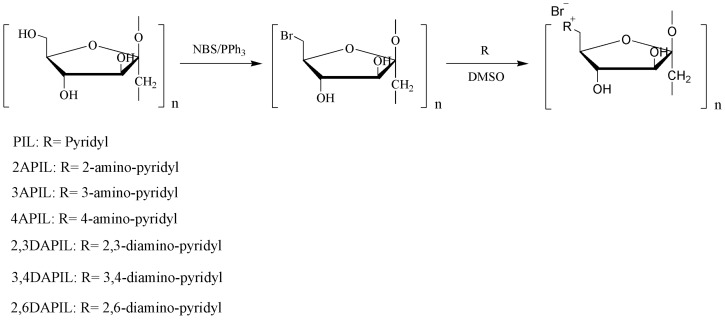
Synthetic pathway of inulin derivatives.

The main procedure showed in Scheme 1 was conducted according to previous methods with appropriate adjustments [42]. A total of 4.83 g (10 mmol) inulin dissolved in 100 mL DMF was added NBS (30 mmol) with magnetic stirring. After that, an ice bath was used to reduce the temperature to about 0 °C. Ph_3_P (30 mmol) was subsequently added in batches within 30 min. The solution was reacted at 80 °C under an argon shield for 3 hr. As soon as the solution cooled to room temperature (RT), we poured it into 800 mL of acetone, and a great quantity of precipitate was formed; the precipitate then was separated by suction filtration and washed with acetone, and freeze dried at −50 °C under vacuum for further reactions. Yield: 92%.

#### 2.2.2. Synthesis of Cationic Inulin Derivatives Bearing Pyridine or Amino-Pyridine Groups

Briefly, 0.45 g (2 mmol) 6-bromo-6-deoxyinulin was mixed with pyridine (3.3 mL, 40 mmol) or amino-pyridine (40 mmol), with 25 mL DMSO as the solvent. The mixture was mildly stirred at 80 °C for 24 h. When the solution was cooled to room temperature, it was poured into a dialysis bag (500 g/mol, molecular weight cutoff (MWCO), prewet with water) that was placed in a beaker with 500 mL ethyl alcohol; the mixture were dialyzed for the first two days in ethyl alcohol, and for another two days in 1000 mL of distilled water. At last, the product was obtained by freeze-drying at −50 °C under a vacuum. Yield: 44–56%.

### 2.3. Analytical Methods

#### 2.3.1. Fourier Transform Infrared Spectroscopy (FT-IR) Spectroscopy

The infrared spectrum was recorded at room temperature in the range of 400–4000 cm^−1^ by a Jasco-4100 infrared spectrometer (provided by JASCO Co., Ltd., Shanghai, China).

#### 2.3.2. Nuclear Magnetic Resonance (NMR) Spectroscopy

NMR spectroscopy outputs (AVIII-500, Berne, Switzerland, provided by Bruker Tech. and Serv. Co., Ltd., Beijing, China) of samples were recorded in the range of 0–10 ppm (^1^H NMR spectra) and 0–180 ppm (^13^C NMR spectra) at room temperature.

#### 2.3.3. Ultraviolet (UV) Spectroscopy

The ultraviolet-visible (UV-vis) spectrum was recorded on a UV-vis spectrometer (TU-1810, provided by Puxi General Instrument Co., Ltd., Beijing, China,).

### 2.4. Water Solubility

The test of water solubility was carried out according to our previous methods with appropriate modification [14]. A total of 0.5 g inulin or inulin derivatives were stirred in 0.5 mL of distilled water at 25 °C until the dissolution equilibrium was reached. Then, the undissolved parts were separated by gravity filtration, subsequently washed with diethyl ether, and freeze dried at −50 °C thoroughly under a vacuum. The test was performed in triplicate and the values were expressed as mean ± the standard deviation (*SD*, *n* = 3). The water solubility (*S*) of inulin and its derivatives were calculated with the following Equation (1):

*S* = (500−*m*_1_)/2 (1)

where *m*_1_ means the weight of the undissolved parts (mg).

### 2.5. Radical Scavenging Assays

The radical scavenging assays (hydroxyl-radical scavenging activity and DPPH-radical scavenging activity) were conducted according to the literature [43,45].

#### 2.5.1. Hydroxyl Radicals’ Scavenging Capacities

Fenton oxidization was utilized to test the scavenging effect against the hydroxyl-radical [43]. All of the solutions were prepared, as follows: Phosphate buffer solution was prepared: 41.58 g Na_2_HPO_4_·12H_2_O and 5.289 g NaH_2_PO_4_·2H_2_O were accurately weighed and dissolved in distilled water, and were then made up to a constant volume of 1000 mL in a volumetric flask. Secondly, 55.6 mg FeSO_4_·7H_2_O and 148.9 mg ethylenediamine tetraacetic acid disodium salt (EDTA–2Na) dissolved by distilled water were mixed, and were made up to a constant volume of 100 mL with a volumetric flask, to obtain the EDTA–Fe^2+^ solution. Thirdly, 36 mg safranin O was accurately weighed and dissolved in the aforementioned phosphate buffer solution to a constant volume to 100 mL in a volumetric flask. At last, 3% H_2_O_2_ solution was prepared using the aforementioned phosphate buffer solution. All of the above solutions were fresh and photophobic. Inulin or its derivatives (PIL, 2APIL, 3APIL, 4APIL, 2,3DAPIL, 3,4DAPIL, or 2,6DAPIL) were dissolved in distilled water and were prepared at concentrations of 0.45, 0.9, 1.8, 3.6, or 7.2 mg/mL.

In a test tube, 1 mL of inulin or an aqueous solution of its derivative, 0.5 mL of EDTA–Fe^2+^ solution, 1 mL of potassium phosphate buffer solution, 1 mL of safranin O solution, and 1 mL of H_2_O_2_ solution were added in order. After the mixture was incubated at 37 °C for 30 min, its ultraviolet absorption at 520 nm was measured. The blank sample consisted of 1 mL of distilled water instead of the sample solution. Three replicates for each sample were tested and the hydroxyl radical scavenging activity was calculated according to the following Equation (2):

(2) $$\mathrm{Scavenging}\;\mathrm{effect}\;\left(\%\right)=\frac{A_{\mathrm{sample}}-A_{\mathrm{blank}}}{A_{\mathrm{control}}-A_{\mathrm{blank}}}$$

where *A*_control_ is the absorbance at 520 nm of the control (distilled water instead of H_2_O_2_) and *A*_blank_ is the absorbance at 520 nm of the blank (distilled water instead of the samples).

#### 2.5.2. DPPH Radicals’ Scavenging Activities

The main process for evaluating the DPPH radicals’ scavenging effect on the derivatives was based on our previous methods [14]. A total of 35.49 mg DPPH was accurately weighed and dissolved in absolute ethyl alcohol, and then made up to a constant volume of 500 mL with a volumetric flask. After that, 1 mL of the testing sample dissolved in distilled water was mixed with 2 mL of the DPPH mentioned above to make test concentrations of 0.1, 0.2, 0.4, 0.8, and 1.6 mg/mL, and these were incubated for 30 min at RT. The entire experimental process was conducted under photophobic conditions to ensure the accuracy of the analysis results. Finally, we tested the ultraviolet absorption at 520 nm; the control group was tested with ethanol instead of DPPH solution and the blank group was tested with 1 mL of distilled water instead of sample solution. Three replicates for each sample were tested and the DPPH-radical scavenging effect was calculated according to the following Equation (3):

(3) $$\mathrm{Scavenging}\;\mathrm{effect}\;\left(\%\right)=[1-\frac{A_{\mathrm{sample}}-A_{\mathrm{control}}}{A_{\mathrm{blank}}}]\times 100$$

where *A*_sample_ is the absorbance of the sample (with DPPH) at 517 nm; *A*_control_ is the absorbance of the control (ethanol instead of DPPH) at 517 nm; and, *A*_blank_ is the absorbance of the blank (distilled water instead of sample solution) at 517 nm.

## 3. Results and Discussion

### 3.1. Structures of Inulin and Cationic Inulin Derivatives Bearing Pyridine or Amino-Pyridine Groups

**Figure 1:**
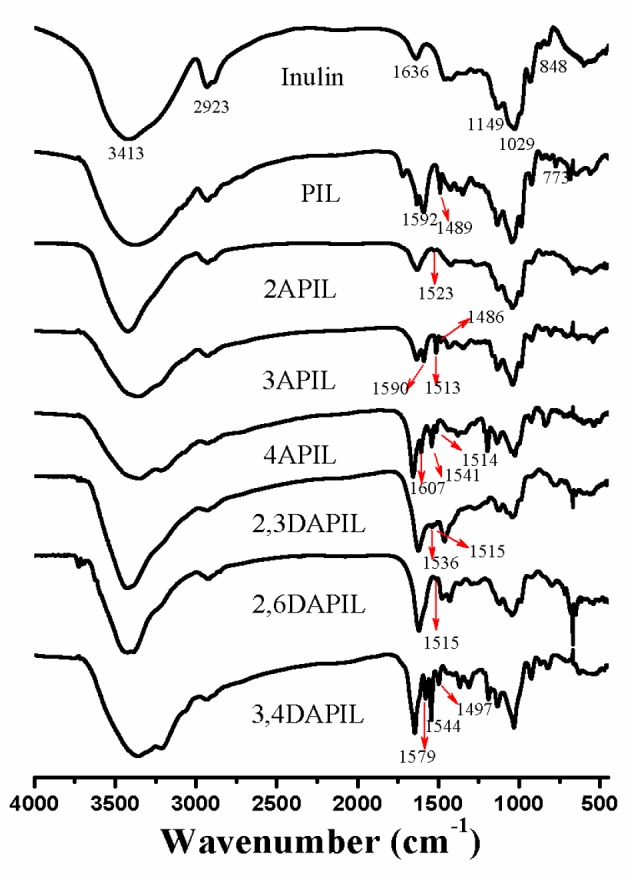
Fourier Transform Infrared Spectroscopy (FT-IR) spectra of inulin and cationic inulin derivatives.

**Figure 2:**
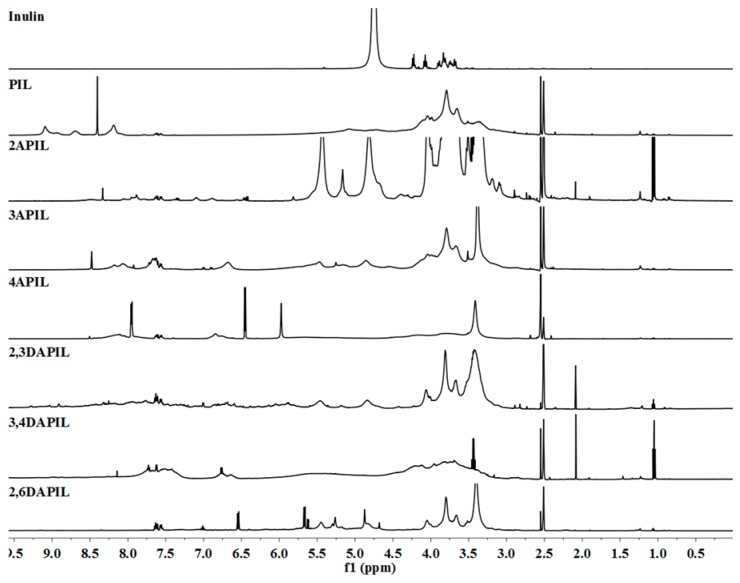
^1^H NMR spectra of inulin and cationic inulin derivatives.

**Figure 3:**
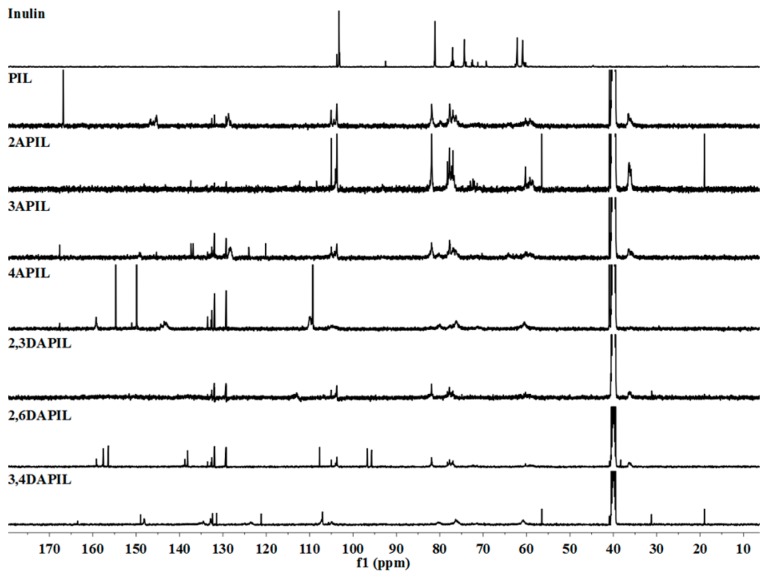
^13^C NMR spectra of inulin and cationic inulin derivatives.

The FT-IR spectra (Figure 1), ^1^H NMR spectra (Figure 2), and ^13^C NMR spectra (Figure 3) of inulin and its derivatives are recorded as the following figures.

Figure 1 shows the FT-IR spectra of inulin and inulin derivatives. Accordingly, the absorption at 3413, 1029, and 848 cm^−1^ belonged to the characteristic absorption bands of inulin. After pyridine was grafted onto inulin, the new absorption values at about 773, 1489, and 1592 cm^−1^ could be attributed to the characteristic absorbance of pyridine. As for the spectra of 2APIL, 3APIL, 4APIL, 2,3DAPIL, 2,6DAPIL, and 3,4DAPIL, the peak between 1480 and 1600 cm^−1^ could be considered as the characteristic absorption peak of the amino-pyridine group.

NMR spectroscopy could be used to characterize the structure of the derivatives more clearly. As shown in Figure 2 and Figure 3, the signals of the inulin molecule were located at 3.0–5.4 ppm (^1^H NMR spectra) and at 60–100 ppm (^13^C NMR spectra). After characterization, the signals of inulin were still existent. For the ^1^H NMR spectrum of PIL, signals at 8.2, 8.7, and 9.1 ppm belonged to five CH protons in the pyridinium ring. Similarly, signals at 129, 132, and 145 ppm in the ^13^C NMR spectra further confirmed the modification with pyridinium. As for the inulin derivatives that were modified with amino-pyridine, these signals moved to the high field at about 6.0–8.5 ppm (^1^H NMR spectra) in 2APIL, 3APIL, 4PAIL, 2,3DAPIL, 3,4DAPIL, and 2,6DAPIL, which should be related to the conjugate structure. The ^13^C NMR spectra in Figure 3 also clearly showed the corresponding signals of amino-pyridine at 95–160 ppm. As a result, inulin has been proven to be successfully modified chemically by pyridine and amino-pyridine.

### 3.2. Water-Solubilities of Inulin and Inulin Derivatives

**Figure 4:**
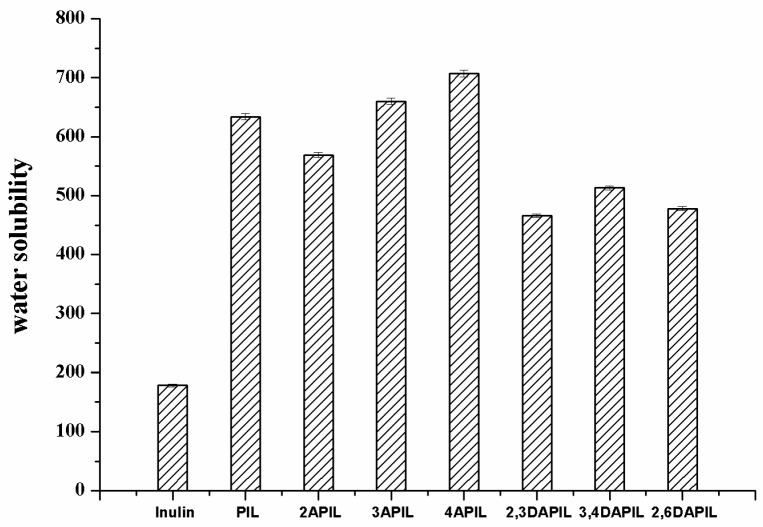
Water solubility of inulin and cationic inulin derivatives

As mentioned, inulin is a kind of water-soluble dietary fiber, and its water solubility is significantly affected by the degree of polymerization and the temperature of the aqueous solution. The values are shown in Figure 4. These reveal that the water solubility of inulin at 25 °C is about 178 mg/mL. All of the cationic inulin derivatives exhibited better water solubility when compared with inulin, and the enhanced solubility is probably due to the cationic modification.

### 3.3. Radical Scavenging Activity

**Figure 5:**
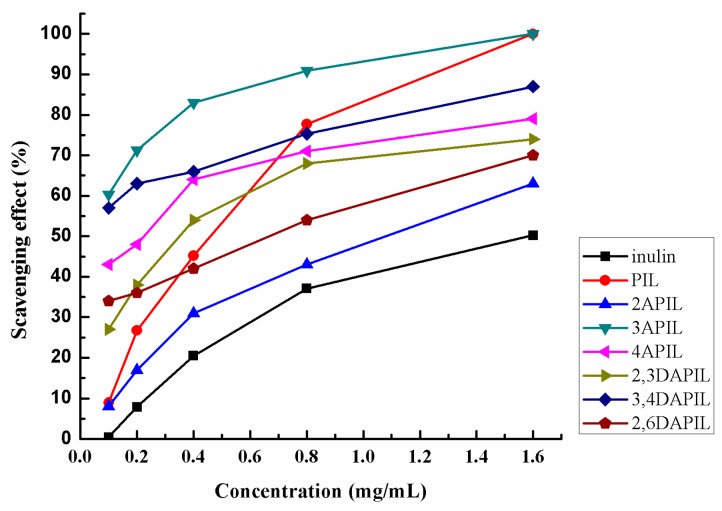
Hydroxyl-radical scavenging abilities of inulin and cationic inulin derivatives.

**Figure 6:**
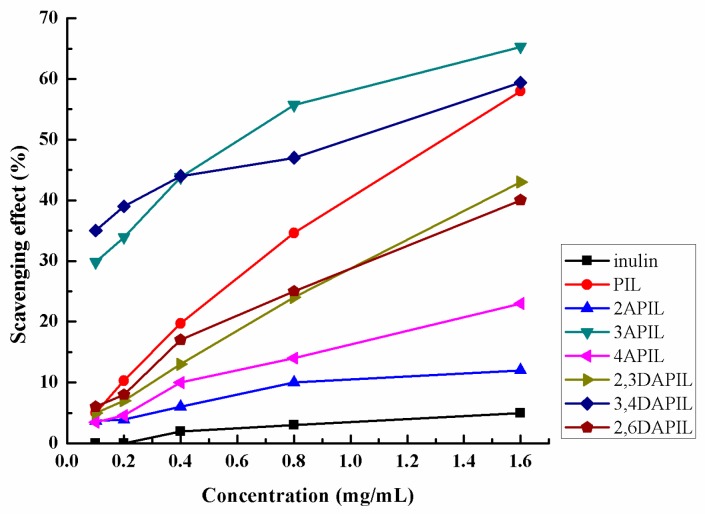
Diphenylpicryl phenylhydrazine (DPPH)-radical scavenging abilities of inulin and cationic inulin derivatives.

The radical scavenging activity (hydroxyl radicals’ scavenging activity and the DPPH radicals’ scavenging activity) of inulin and of all of the cationic inulin derivatives are shown in the following figures (Figure 5 and Figure 6). A short discussion of the relationship between their structures and the radical scavenging activities of the inulin derivatives are presented subsequently.

As is shown in Figure 5, inulin without any modification showed the lowest hydroxyl-radical scavenging ability, but the clearance rate could reach 50% at 1.6 mg/mL, which should be related to the multiple hydroxyl groups of inulin. The result reveals the potential of inulin as a natural antioxidant. Then, after being modified with the pyridine and amino-pyridine group, inulin exhibited an obvious improvement on scavenging of the hydroxyl-radical. It also showed a positive correlation between the scavenging ability and the concentrations of all of the samples. The hydroxyl-radical scavenging ability decreased in the following order: 3APIL > PIL > 3,4DAPIL > 4APIL > 2,3DAPIL > 2,6DAPIL > 2APIL. Among them, 3APIL showed the best scavenging ability and it could completely scavenge the hydroxyl-radical at 1.6 mg/mL. This result was consistent with the earlier report that amino conjugated at the 3-C position of pyridine had strong biological activity effects in vitro [46]. For PIL, it also showed an excellent hydroxyl-radical scavenging effect, and it was more sensitive to its concentration. Like 3APIL, PIL could also completely clear hydroxyl radicals at 1.6 mg/mL, which should be attributed to the high positive charge density and it should also be free from steric hindrance. As a consequence of the steric hindrance, amino that was grafted on the *ortho*-position of pyridine always showed a lower hydroxyl-radical scavenging activity. 

The scavenging ability of inulin and of the synthesized products against the DPPH radical are shown in Figure 6. It is observed that inulin itself had no effect on scavenging DPPH radicals. In contrast, all of the derivatives exhibited an improvement on the scavenging effect, and the effect had a positive correlation with the tested concentrations. The DPPH-radical scavenging ability decreased in the following order: 3APIL > 3,4DAPIL > PIL > 2,3DAPIL > 2,6DAPIL > 4APIL > 2APIL. This was similar to the hydroxyl-radical scavenging activity, in that 3APIL showed the best effect on DPPH-radical scavenging. 2APIL had the lowest DPPH-radical scavenging activity, due to high steric hindrance. 2,3DAPIL and 2,6DAPIL exhibited better DPPH-radical scavenging activity than 2APIL, which was possible due to the greater frequency of amino grafting.

## 4. Conclusions

Many natural saccharides that were extracted from plants have exhibited excellent antioxidant properties, which can be developed as novel potential antioxidants. Chemical modification plays an important role in further improving the biological activities of these saccharides. In this article, inulin is designed to be modified by pyridine and a series of amino-pyridine groups, in order to study how these groups affect the bioactivity of inulin. The synthetic method is simple and efficient. The result shows that all derivatives reveal an enhancement on water-solubility when compared with inulin, and that their radical scavenging activities are also improved distinctly, which proves that the introduction of the amino group, as well as the increased density of the positive charge of inulin, could help to enhance its biological activity. Amino conjugated at the 3-C position of pyridine exhibits a strong bioactivity effect. The steric hindrance effect and substitute position of the amino group is the key factor that affects the radical scavenging activity of the synthesized inulin derivatives. Further comprehensive investigations in ascertaining this hypothesis on radical scavenging activity and structure–activity relationships will be carried out.
